# Precision installation of a highly efficient suicide gene safety switch in human induced pluripotent stem cells

**DOI:** 10.1002/sctm.20-0007

**Published:** 2020-07-13

**Authors:** Zhong‐Dong Shi, Jason Tchao, Ling Wu, Aaron J. Carman

**Affiliations:** ^1^ InVitro Cell Research, LLC Greater New York Metropolitan Area New York USA

**Keywords:** AAVS1 locus, CAG promoter, genome editing, genomic safe harbor, induced pluripotent stem cell, inducible caspase‐9, safety switch, stem cell therapy, suicide gene

## Abstract

Human pluripotent stem cells, including induced pluripotent stem cells (iPSCs) and embryonic stem cells, hold great promise for cell‐based therapies, but safety concerns that complicate consideration for routine clinical use remain. Installing a “safety switch” based on the inducible caspase‐9 (iCASP9) suicide gene system should offer added control over undesirable cell replication or activity. Previous studies utilized lentiviral vectors to integrate the iCASP9 system into T cells and iPSCs. This method results in random genomic insertion of the suicide switch and inefficient killing of the cells after the switch is “turned on” with a small molecule (eg, AP1903). To improve the safety and efficiency of the iCASP9 system for use in iPSC‐based therapy, we precisely installed the system into a genomic safe harbor, the *AAVS1* locus in the *PPP1R12C* gene. We then evaluated the efficiencies of different promoters to drive iCASP9 expression in human iPSCs. We report that the commonly used EF1α promoter is silenced in iPSCs, and that the endogenous promoter of the *PPP1R12C* gene is not strong enough to drive high levels of iCASP9 expression. However, the CAG promoter induces strong and stable iCASP9 expression in iPSCs, and activation of this system with AP1903 leads to rapid killing and complete elimination of iPSCs and their derivatives, including MSCs and chondrocytes, *in vitro*. Furthermore, iPSC‐derived teratomas shrank dramatically or were completely eliminated after administration of AP1903 in mice. Our data suggest significant improvements on existing iCASP9 suicide switch technologies and may serve as a guide to other groups seeking to improve the safety of stem cell‐based therapies.

## Graphical abstract

Therapeutic cells made from induced pluripotent stem cells (iPSCs) have many clinical applications but carry risks. Our improved “suicide switch” is designed to eliminate potentially dangerous cells and protect patients.

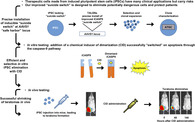

**Significance statement**

With the growth of human induced pluripotent stem cell (iPSC)‐derived cell therapies, the need for effective safety measures increases. There is an urgent need for improved, inducible cell “suicide” systems that encompass a diversity of “keys” (CIDs) and “locks” (inducible apoptotic mechanisms). Currently, only a few unique systems exist. This study describes the improved design, in vitro and in vivo testing of a novel inducible “suicide switch” safety system for iPSC‐derived cell‐based therapies.

## 1 INTRODUCTION

Due to their extensive self‐renewal potential and ability to differentiate into virtually any cell type in the human body, induced pluripotent stem cells (iPSCs) hold great promise for regenerative medicine.^1^, ^2^ However, engineering cells from iPSCs for use as therapies in humans poses several notable risks: residual undifferentiated iPSCs may form teratomas after transplantation, genetic or epigenetic aberrations from cellular reprogramming or prolonged cell culture may increase oncogenic potential of therapeutic cells, and cell products derived from iPSCs may have unforeseen toxic activity.^3^, ^4^, ^5^, ^6^ Thus, the incorporation of a highly efficient safety switch that can be activated to selectively eliminate aberrant therapeutic cells that itself does not introduce more risk is desirable. Such inducible genetic switches are referred to as “suicide” gene safety switches.

To date, three types of suicide gene technologies have been developed.^7^ Overexpression of CD20 allows cells to be targeted by administration of an anti‐CD20 monoclonal antibody and eliminated through complement/antibody‐dependent cellular cytotoxicity.^8^ However, endogenous CD20‐expressing cells may be inadvertently killed with this method, leading to potentially toxic off‐target effects. A metabolic suicide gene system using the herpes simplex virus thymidine kinase (HSV‐TK) gene and its prodrug, ganciclovir, has been widely discussed.^9^ HSV‐TK initiates a phosphorylation cascade of ganciclovir that yields a product that competes with deoxyguanosine triphosphate for incorporation into replicating DNA, causing cell death. HSV‐TK has been tested in human iPSCs as a suicide gene system.^10^, ^11^ However, one recent study showed that when the HSV‐TK was knocked into a genomic safe harbor on chromosome 1, it was able to kill the iPSCs in vitro, but not sufficient to eliminate or shrink iPSC‐derived teratomas significantly in vivo.^12^ In addition, ganciclovir is a potent cytotoxic antiviral drug used to treat herpes virus infections; in the event that ganciclovir is needed to treat a viral infection, it will also unavoidably kill transplanted cells expressing the HSV‐TK suicide gene system.^7^ Another potential problem for the use of HSV‐TK as a suicide gene system is that the system may be immunogenic in humans.^13^

Inducible caspase‐9 (iCASP9) was developed as a dimerization‐induced suicide gene system and was demonstrated to be safe and effective in clinical trials.^14^, ^15^, ^16^ The iCASP9 system was engineered by replacing the caspase recruitment domain of pro‐apoptotic caspase‐9, a mitochondrial death pathway molecule, with a mutated dimerizer drug‐binding domain from the human FK506‐binding protein (FKBP12‐F36V).^15^, ^17^ The F36V mutation confers to the fusion protein extremely high affinity for chemical inducers of dimerization (CID) such as AP1903, which induces rapid apoptosis by activating caspase‐9 and downstream effector caspases such as caspase‐3. AP1903 (aka rimiducid) is a lipid‐permeant tacrolimus analogue (C_78_H_98_N_4_O_20_, molecular weight: 1411.6 g/mol). The iCASP9 system previously was installed into T cells and human iPSCs using lentiviral infection.^14^, ^18^, ^19^, ^20^, ^21^ Administration of CIDs can rapidly kill up to 90% to 99% of the cells transduced with iCASP9 and can control human iPSC‐derived teratoma growth in mice.

Although 99% killing efficiency demonstrates a valuable in vitro experimental result, it must be further optimized for clinical use, a setting in which a patient's life may depend on complete elimination of engineered therapeutic cells that become problematic. Additionally, nonspecific lentivirus‐mediated genomic integration of the iCASP9 system is not ideal for clinical cell products because the integration may result in deleterious, especially oncogenic, genetic changes, or unexpected silencing. Indeed, this has been reported in human iPSCs with an iCASP9 system driven by either the EF1α promoter or its core promoter.^19^, ^20^, ^22^

To improve the safety and efficiency of the iCASP9 system in stem cell‐based therapy, we sought to install the iCASP9 system precisely into a known human genome safe harbor. There are several known genome safe harbor sites, including the *AAVS1*, *CCR5*, and *hROSA26* in the human genome; among these, however, only the *AAVS1* locus has been relatively well studied.^23^ This locus resides within the intron 1 of the *PPP1R12C* gene on human chromosome 19.^23^ Genome editing in the *AAVS1* locus has not been reported to result in proliferation or differentiation abnormalities in either embryonic stem cells (ESCs) or iPSCs.^23^, ^24^, ^25^ Transgene expression in this locus driven by the endogenous promoter of the *PPP1R12C* gene is stable and consistent in many cell types.^23^, ^24^ Additionally, no disease has been linked to the disruption of *PPP1R12C* gene, based on previous studies. These characteristics make the *AAVS1* locus a potentially ideal location for iCASP9 installation for clinical use. We also evaluated efficiencies of several promoters to drive iCASP9 expression in human iPSCs, including the EF1α promoter, the endogenous promoter of the *PPP1R12C* gene, and the CAG promoter. We demonstrate that among the tested promoters, the CAG promoter gives stable and strong transgene expression and that, upon treatment with AP1903, the iPSC clones that contain two copies of iCASP9 and their derivatives can be efficiently killed in vitro and iPSC‐derived teratomas can be eliminated or significantly shrunk in vivo.

## 2 MATERIALS AND METHODS

### 2.1 Human iPSC culture

Human iPSC (clone m26) was generated in‐house from renal epithelial cells of an apparently healthy male using the Simplicon mRNA reprogramming kit (Millipore Sigma, Cat. SCR550). Prior to reprogramming, the renal epithelial cells were maintained on gelatin coated surfaces in RE/MC proliferation medium consisting of a 1:1 mixture of Renal Cell Growth Medium/REGM SingleQuots (Lonza, Cat. CC‐3190) and DMEM high glucose supplemented with 10% FBS, 1% Glutamax, 1% non‐essential amino acids, 5 ng/mL bFGF, 5 ng/mL PDGF, and 5 ng/mL EGF (all from Gibco). Human iPSCs were cultured in Essential 8 Flex (E8 Flex) medium (Thermo Fisher Scientific, Cat. A2858501) with vitronectin (Thermo Fisher Scientific, Cat. A14700) as the coating substrate. Cells were maintained at 37°C with 5% CO_2_, and regularly confirmed to be mycoplasma free. Cells were passaged every 3 to 4 days using Versene (Thermo Fisher Scientific, Cat. 15040066) for dissociation. A 5 μM Rho‐associated protein kinase inhibitor (ROCKi) Y‐27632 (Selleck Chemicals, Cat. S1049) was only added into the culture media for the first day when passaging or thawing iPSCs unless otherwise stated. Karyotypes were regularly checked by the Molecular Cytogenetics Core at Memorial Sloan Kettering Cancer Center.

### 2.2 Vector construction and viral production

The *AAVS1* targeting donors were designed by InVitro Cell Research, LLC and synthesized and cloned by IDT (Coralville, Iowa) and GenScript (Piscataway, New Jersey). The AAVS1 TALEN constructs were kindly provided by Dr Danwei Huangfu at Memorial Sloan Kettering Cancer Center. The CAG‐Luciferase‐GFP construct was custom designed and the cloning and lentivirus production were performed by Biosettia (San Diego, California).

### 2.3 Nucleofection and stable line generation

The AAVS1 TALEN and donor constructs were delivered to the iPSCs via nucleofection using 4D Nucleofector X Unit (Lonza) and P3 Primary Cell X kit L (Lonza, Cat. V4XP‐3024). Before nucleofection, normal cultured iPSCs at day 4 were treated with 2.5 μM ROCKi for 4 hours and then dissociated with TrypLE Select (Thermo Fisher Scientific, Cat. 12563029) by incubating for 4 minutes at room temperature. Cells were collected, counted, and then pelleted at 130*g* for 5 minutes. The pellet was resuspended into P3 solution with supplement (10 million cells for each 100‐μL nucleofection), followed by mixing with TALEN and donor plasmids (5 μg TALEN‐L and 5 μg TALEN‐R, either with 12 μg donor for single donor experiments, or with 7 μg donor 4‐p plus 7 μg donor 4‐n for dual selection experiments). The total volume of plasmid mixtures was within 10% of nucleofection volume. Cells were immediately subjected to nucleofection using program CA137 and plated into vitronectin‐precoated 10‐cm cell culture dishes (Corning, Cat. 353003) containing 10 mL pre‐warmed E8 Flex medium and 5 μM ROCKi. The whole process was done very gently and quickly to avoid cell damage. Medium was changed the next day to remove ROCKi and dead cells.

**FIGURE 1:**
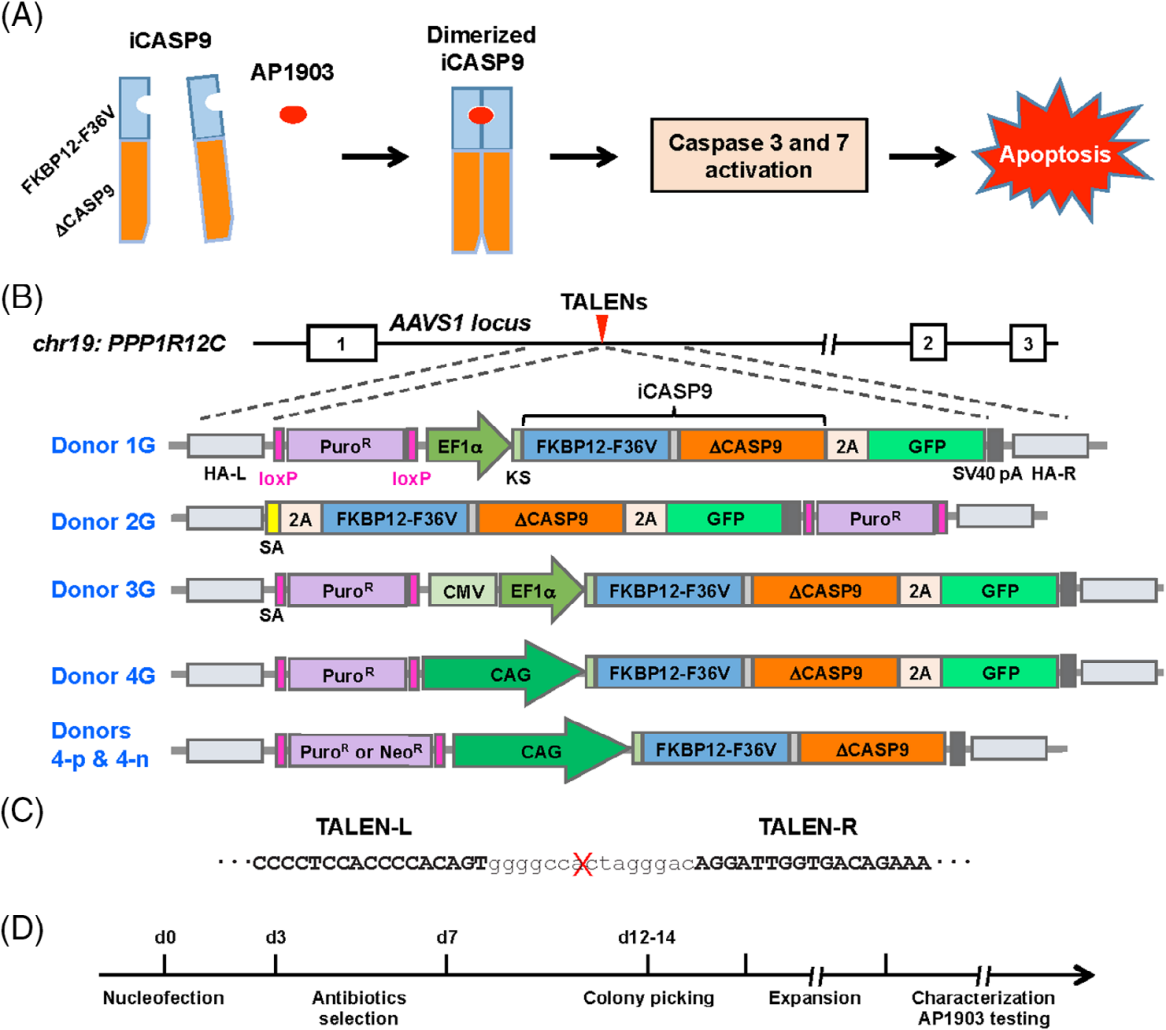
Design and *AAVS1* targeting for installation of iCASP9 safety switch in human induced pluripotent stem cells (iPSCs). A, Components of the iCASP9 system and dimerization of iCASP9 induced by AP1903 activates mitochondrial apoptosis pathway (caspase 3 and 7), leading to apoptotic cell death. B, The *AAVS1* locus is located in the first intron of the *PPP1R12C* gene in human chromosome 19. TALENs are used to create DNA double‐strand breaks for inserting iCASP9 transgenes into the genome via homologous recombination. A total of six donor repair templates were constructed as follows. 1G: Puro‐EF1α‐iCASP9‐2A‐GFP; 2G: SA‐2A‐iCASP9‐2A‐GFP‐Puro; 3G: Puro‐CMV‐EF1α‐iCASP9‐2A‐GFP; 4G: Puro‐CAG‐iCASP9‐2A‐GFP; 4‐p: Puro‐CAG‐iCASP9; and 4‐n: Neo‐CAG‐iCASP9. EF1α, EF1α core promoter; HA‐L and HA‐R, left and right homology arms. SV40 late poly(A) was used in all the constructs and located right after the last transgene; KS, Kozak sequence; CMV, CMV enhancer; SA, splicing acceptor. PuroR or NeoR spanned by two loxP sites were used for puromycin or G418 selection during clonal line establishment. The expression of PuroR and NeoR were driven by the SV40 promoter and poly(A) tail was the synthetic poly(A). The GFP versions of the donor templates were used to quickly assess iCASP9 expression levels based on GFP levels. C, The sequences of the left and right *AAVS1* TALENs and their target site in the *AAVS1* locus. D, Clonal line production: from nucleofection to characterization

Antibiotics selection started at day 3 after nucleofection with 0.5 μg/mL puromycin and/or 75 μg/mL G418 for single or dual selection (Figure 1D).

Antibiotic‐resistant clones were picked between day 12 and day 14 after nucleofection and mechanically disaggregated and replated into individual wells of 96‐well plates (Corning, Cat. 353072) precoated with vitronectin containing E8 flex medium and ROCKi. Clonal lines were then expanded at day 4 or day 5.

### 2.4 Clonal line characterization

During clonal line expansion, a portion of the cells was lysed by 1 mg/mL proteinase K (Roche, Cat. 50‐720‐3027) in 1X JumpStart PCR buffer (Sigma, Cat. P2192) overnight at 56°C to release genomic DNA followed by 10 minutes at 99°C to inactivate proteinase K as previously described.^26^ Then real‐time PCR was performed using qBiomarker SYBR ROX FAST Mastermix (Qiagen, Cat. 337840) to calculate copy numbers of iCASP9 in each picked clone. The PCR was run on the StepOnePlus system (Applied Biosystems) using the following protocol: 2 minutes at 50°C and 2 minutes at 95°C followed by 45 cycles of 15 seconds at 95°C and 1 minute at 60°C. The signal was detected at 60°C. The dissociation curve was also performed to confirm a single peak from reactions. The primer sequences are iCASP9‐F:TGTGGGTCAGAGAGCCAAAC, iCASP9‐R: CAAATCTGCATTTCCCCTCA; and GAPDH‐F: CATGCCTTCTTGCCTCTTGTCTCTTAGAT, GAPDH‐R: CCATGGGTGGAATCATATTGGAACATGTAA, which have been published previously.^20^ Some clones were further expanded and then characterized by karyotyping (Molecular Cytogenetics Core Facility, Memorial Sloan Kettering Cancer Center, New York, New York) and whole genome sequencing (Genewiz, South Plainfield, New Jersey).

### 2.5 In vitro AP1903 treatment and cell death measurement on iPSCs

iPSCs were cultured in E8 Flex medium for 2 to 4 days in 24‐well, 12‐well, and/or 6‐well plates (Corning) when cell density reached ∼0.15 × 10^6^/cm^2^ and then exposed to 10 or 50 nM AP1903 (Medchemexpress, Cat. HY‐16046) for the desired amount of time. All cells in each well at the time point were collected and stained with annexin V (AnV) and 7‐amino‐actinomycin D (7‐AAD) (Enzo Life Sciences, Cat. ENZ‐51002‐100) for 15 minutes following the manufacturer's instructions. Cells were then quantified and analyzed by flow cytometry (Attune NxT, Thermo Fisher Scientific) and Flowjo Software. The dead cells were quantified using 100% minus the percent AnV^−^/7‐AAD^−^ cells in the FSC/SSC gated region after doublet removal. The real percentage of total dead cells in the AP1903 treated condition at some time points could be even higher since majority of the cells became debris and were not included in calculation (Figure 3E,F).

### 2.6 MSC and chondrogenic differentiation and AP1903 treatment

The human iPSC clone containing iCASP9 (4‐pn‐45) was differentiated into mesenchymal stem cells (MSCs) using Stem Cell Technologies Stemdiff Mesenchymal Progenitor Kit according to the manufacturer protocol. iCASP9 MSCs were expanded on Cellstart CTS (Gibco) coated flasks at 1:100 dilution and Mesencult ACF Plus (Stem Cell Technologies). Flow cytometry was used to characterize cell phenotype and purity. CD44, CD73, and CD105 antibodies were purchased from Stem Cell Technologies (Cat. 60068AD, 60044FI, 60039AD, respectively). At passage 7, the MSCs were treated with AP1903 for 0, 1, 4, and 24 hours and cells were harvested by TrypLE digestion followed by Trypan blue staining. Live and dead cells were counted by Countess II (Thermo Fisher Scientific). Cell morphology change was also assessed by microscopy at different time points.

For micromass chondrogenic differentiation, a protocol was derived from a previously published method.^27^ MSCs cultured in monolayers were harvested by TrypLE, and resuspended in type I Collagen Gel (Advanced BioMatrix) at a density of 1 × 10^7^ viable cells/mL. Then, 10 μL of cell‐gel suspension was dipped on the plastic surface of a 6‐well plate. Gelation was initiated by incubation at 37°C for 1 hour. Then, 2 mL of chondrogenic differentiation medium (STEMPRO Chondrogenesis Differentiation Kit, Thermo Fisher Scientific) was added to each well. Micromass was cultured for 1 week before fixing with 10% formalin. Alcian blue staining was performed, followed by PBS wash. For live/dead assessment, AP1903 was added to the medium 1 week after chondrogenic differentiation. At 0, 1, 24, and 48 hours after drug treatment, cells were harvested from micromass by TrypLE digestion, followed by Trypan blue staining. Live and dead cells were counted by Countess II (Thermo Fisher Scientific). Cell morphology change was also assessed by microscopy at different time points.

### 2.7 Luciferase labeling, teratoma formation, and killing assay

For in vivo teratoma study, the CAG‐iCASP9 iPSC clones were labeled with lentiviral particle expressing luciferase (CAG‐Luc‐GFP‐IRES‐Hygro, custom designed, produced by Biosettia), followed by single cell subcloning to establish iPSC‐luciferase clonal lines.

Teratoma formation and killing assays were performed at Invivotek LLC (Hamilton, New Jersey), following the protocols and regulations approved by the Institutional Animal Care and Use Committee and the Association for Assessment and Accreditation of Laboratory Animal Care of Invivotek. To form teratomas, iPSC pellets were harvested and gently resuspended with DMEM/F12 medium at 30 million/mL, and 50 μL (1.5 million) of cells mixed with 50 μL Matrigel were injected subcutaneously into the right & left thighs of 5‐week old male NSG mice (Jackson Laboratory). Teratoma formation was monitored using bioluminescence imaging (BLI). After 34 days, the BLI values reached approximately 10^9^ photons/s. Then, the mice were administrated 2 mg/kg AP1903 IP in Solutol (diluted from 5 mg/mL in 25% Solutol stock) or Solutol only as vehicle once a day for five consecutive days. We found that Solutol alone not only had inhibitory effect on teratoma expansion, but also interfered with the function of AP1903. Nine days after AP1903/Solutol treatment, there was no significant difference in the BLI values between AP1903 and Solutol vehicle two groups. On day 62, the mice were regrouped and administered IP or IT 2 mg/kg AP1903 DPT (50% N,N‐dimethylacetamide/50% [90% PEG‐400/10% Tween 80]) for five consecutive days. The BLI values were monitored from day 62 through day 78 and the experiment was terminated at day 83. The percentage changes of the BLI values were calculated. Mice were euthanized once their tumors reached 2000 mm^3^, if the tumors affected their movement or at the end of the experiment.

### 2.8 Statistical analyses

All quantification data are presented as mean ± SD. Cell deaths among different time points were compared using two‐way ANOVA followed by multiple comparison with Tukey correction using Graphpad Prism. *P* < .05 is determined as significant.

## 3 RESULTS

### 3.1 Neither the EF1α promoter nor the endogenous promoter drive stable or strong iCASP9 expression in human iPSCs

To precisely knock‐in the iCASP9 to the *AAVS1* locus, we designed a TALEN‐mediated genome targeting protocol, similar to the strategy previously described.^28^ We designed an *AAVS1* donor construct with the commonly used EF1α core promoter to drive iCASP9 and green fluorescent protein (GFP) expression (Donor 1G). HA‐L and HA‐R are the homology arms for homologous recombination to integrate the transgene into the locus. GFP was used for easy and rapid determination of relative levels of iCASP9 expression. A puromycin resistance marker, which could later be removed by Cre recombinase, was used to select for cells that successfully integrated the iCASP9 transgene construct. Donor templates and TALENs were delivered by nucleofection (Figure 1; Figure S1A).

We generated multiple single‐cell derived clones containing the integrated iCASP9 transgene system after puromycin selection. However, clones derived from transgene insertions driven by the EF1α promoter (Donor 1G) did not express GFP and the cells were not eliminated by the CID, AP1903 (Figure S1A,B). These results suggest that either the EF1α core promoter is not strong enough to drive high levels of iCASP9 expression or that the promoter is silenced in these iPSCs.

**FIGURE 2:**
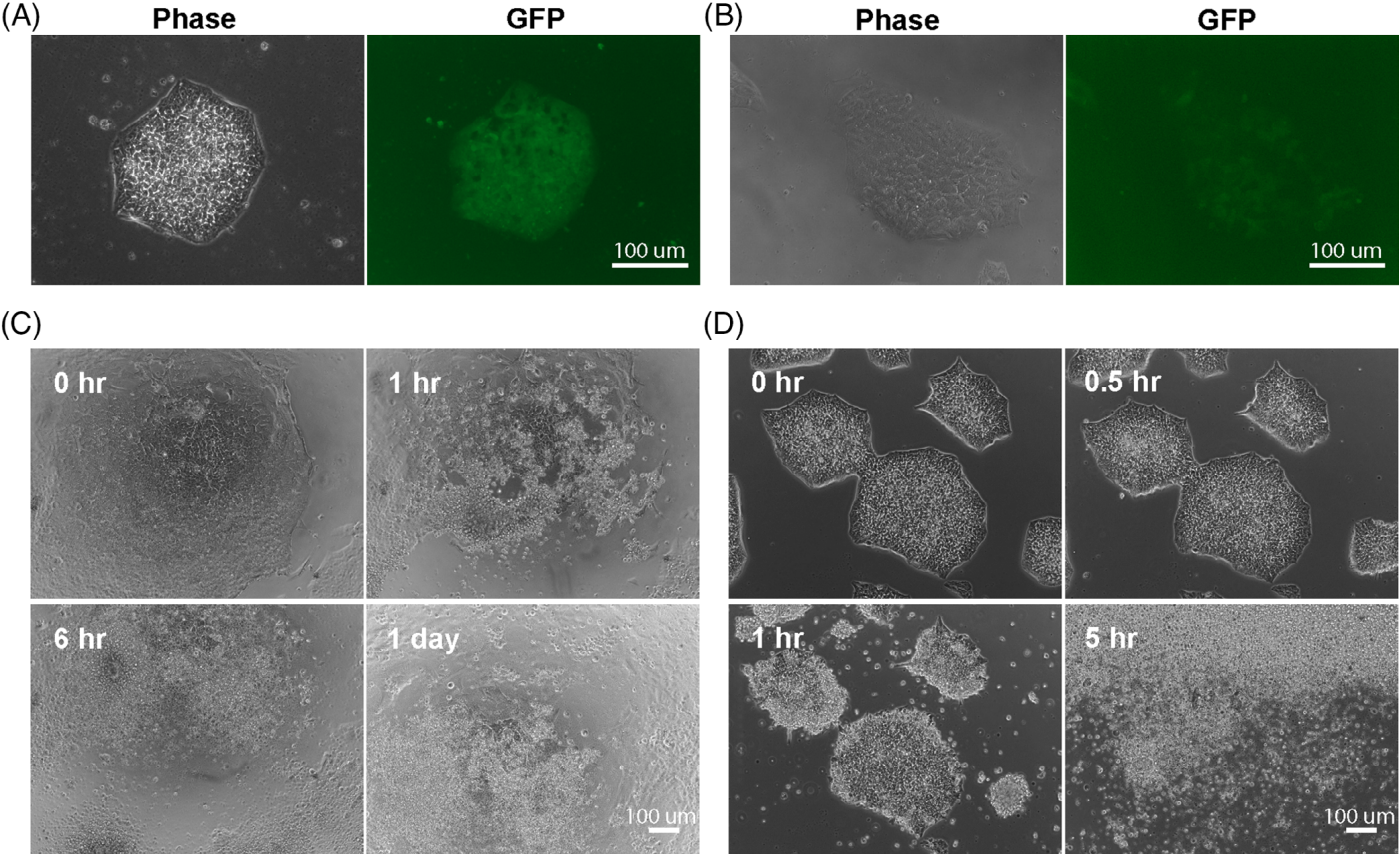
CMV‐EF1α promoter is gradually silenced in the established iCASP9 induced pluripotent stem cells (iPSC) clones. A, Representative images on day 10 after nucleofection: puromycin‐resistant CMV‐EF1α‐iCASP9‐2A‐GFP (Donor 3G) iPSC clones appeared. Clones expressed GFP but some of the cells within the same clone did not express GFP. B, After colony picking, more GFP negative cells appeared. C, Representative images after 24 hours exposure to AP1903 (50 nM) failed to eliminate all cells in the established clones. D, After 72 hours of puromycin reselection, the remaining cells were much more susceptible to AP1903 (50 nM) than before reselection shown in C

We next modified the iCASP9 promoter design to avoid these potential problems and generated Donor 2G that uses the endogenous *PPP1R12C* promoter to provide consistent transgene expression. We also constructed Donor 3G with a CMV enhancer added directly before the EF1α core promoter to enhance transgene expression (Figure 1B). After establishing clonal lines following gene targeting, we discovered that the endogenous promoter (Donor 2G) was insufficient to drive high levels of GFP expression and that the derived clones could only be partially eliminated by AP1903 (Figure S1C,D). Conversely, the CMV‐EF1α promoter (Donor 3G) drove high levels of GFP expression; however, we observed gradual GFP silencing in these clones and incomplete elimination with AP1903 (Figure 2A‐C). This finding is consistent with previous reports that the EF1α promoter can be silenced in human iPSCs.^19^, ^22^, ^29^ After subjecting derived clones to a second round of puromycin selection, the remaining cells were eliminated by AP1903 with significantly greater efficiency (Figure 2D). These data suggest that engineered cells are efficiently eliminated with a transgene suicide system located in the *AAVS1* locus only when a strong promoter that cannot easily be silenced drives the system.

### 3.2 iPSCs containing the iCASP9 system driven by the CAG promoter can be efficiently eliminated by the CID AP1903

**FIGURE 3:**
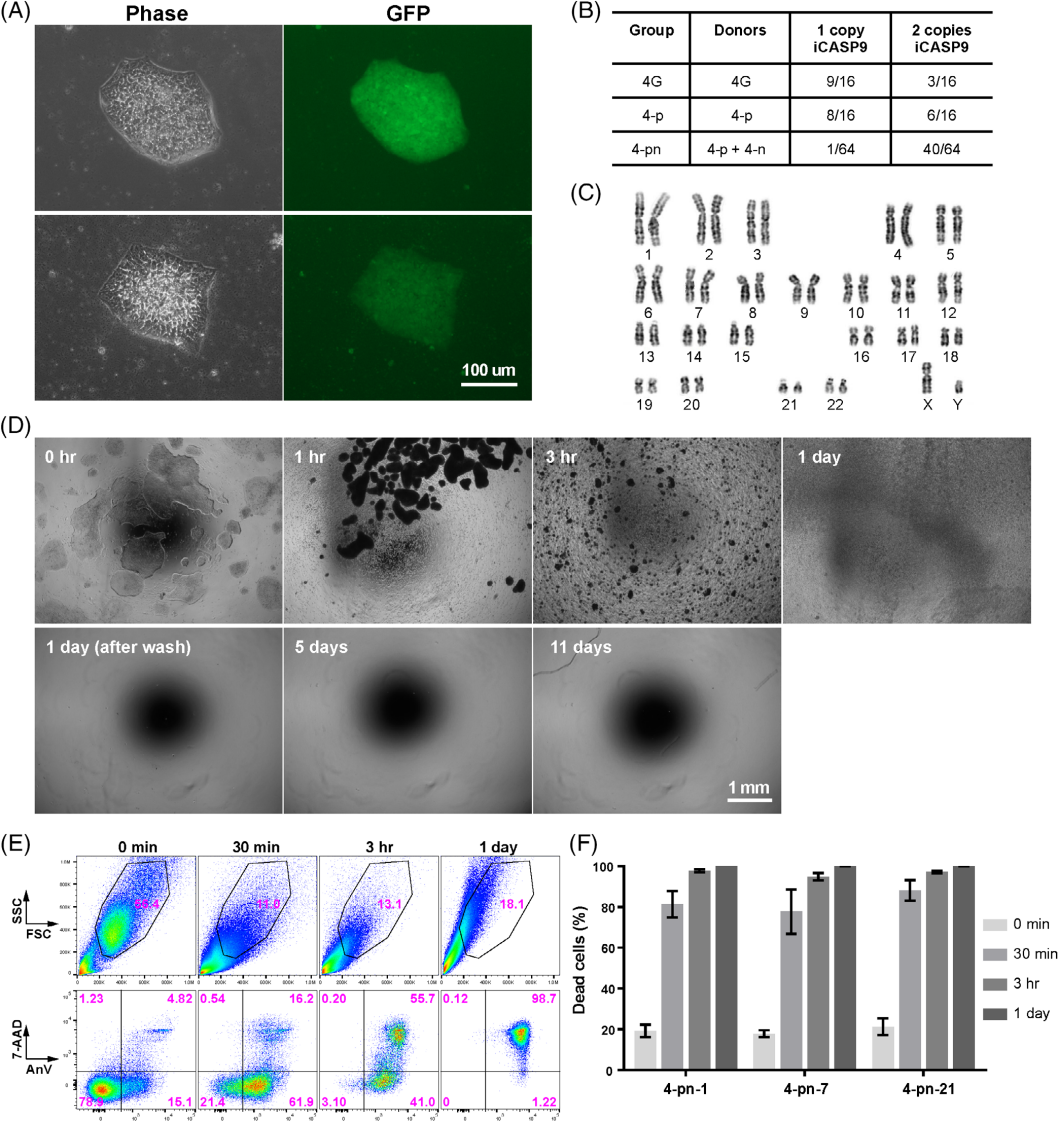
AP1903 rapidly induces apoptosis of the CAG‐iCASP9 induced pluripotent stem cells (iPSCs) in vitro. A, CAG promoter induced uniform, strong and stable GFP expression in the established clones. Representative images from two clones at day 7 post‐nucleofection are shown. B, The number of clones harboring one or two copies of iCASP9 out of the total clones picked in three different targeting groups. C, Karyotypes were normal (46, XY) in all 20 metaphase cells analyzed for each 11 out of total 13 clones examined. D, Representative time course images show AP1903 (10 nM) completely killed CAG‐iCASP9 iPSCs within 24 hours with no cell regrowth. E, Representative time course FACS analyses of cell size and cell death (Annexin V and 7‐AAD) during 10 nM AP1903 treatment. F, Quantification of FACS data from three different iCASP9 iPSC clones. Data are presented as mean ± SD (n = 3‐4). Two‐way ANOVA followed by multiple comparison with Tukey correction was used and *P* < .0001 for all the time points when compared with time 0 minute. The dead cells were quantified using 100% minus the percent AnV^−^/7‐AAD^−^ cells in the FSC/SSC gated region after doublet removal. Therefore, the real percentage of total dead cells in the AP1903 treated condition at some time points could be even higher since majority of the cells became debris and were not included in calculation

In order to improve iCASP9 expression characteristics, we considered selecting a stronger promoter and/or integrating iCASP9 into both alleles of the *AAVS1* locus. We tested the CAG promoter to drive iCASP9 expression and designed three types of donor templates. Donor 4G contained GFP and the puromycin selection marker, whereas Donors 4‐p and 4‐n did not contain GFP but did contain either puromycin (4‐p) or neomycin (4‐n) selection markers (Figure 1B). The non‐GFP versions are rendered more clinically relevant once the antibiotic resistance gene is removed by Cre recombinase. We observed uniform GFP expression in cells successfully integrated with donor template 4G after puromycin selection (Figure 3A) and the GFP expression was stable in these established iPSC clones, suggesting there is no silencing in the CAG promoter. These data also indicate that the loss of transgene expression in our earlier experiment using EF1α promoter was due to silencing of EF1α promoter, not due to loss of transgene integration during culture, as we did not observe a gradual loss of GFP expression. To improve the odds of generating iPSC clones with two copies of iCASP9 (one in each allele at the *AAVS1* locus), the donor templates with puromycin (4‐p) and neomycin (4‐n) were mixed at a 1:1 ratio during nucleofection, followed by double selection with puromycin and neomycin. We obtained the clones with two copies of iCASP9 more efficiently in the case of mixing Donors 4‐p and 4‐n with dual selection (Figure 3B; Figure S2). Based on our earlier observation that higher levels of iCASP9 expression are required for efficient cell elimination, we focused on the clones harboring two copies of CAG‐iCASP9 for further testing. AP1903 (10 nM) treatment induced rapid apoptosis in the iCASP9 iPSCs within 3 hours: cells detached from the plate and became smaller, and more than 95% stained positive for annexin V, a marker of apoptosis (Figure 3D‐F). After 24 hours, the cells were more granulated (Figure S3) and displayed higher side scatter values than at 3 hours by flow cytometry (Figure 3E). AP1903 eliminated all detectable cells within 24 hours and we observed no surviving or expanded cells, even 10 days after the medium had been replaced by fresh medium without AP1903 (Figure 3D‐F).

### 3.3 AP1903 eliminates CAG‐iCASP9 iPSC‐derived MSCs and chondrocytes

**FIGURE 4:**
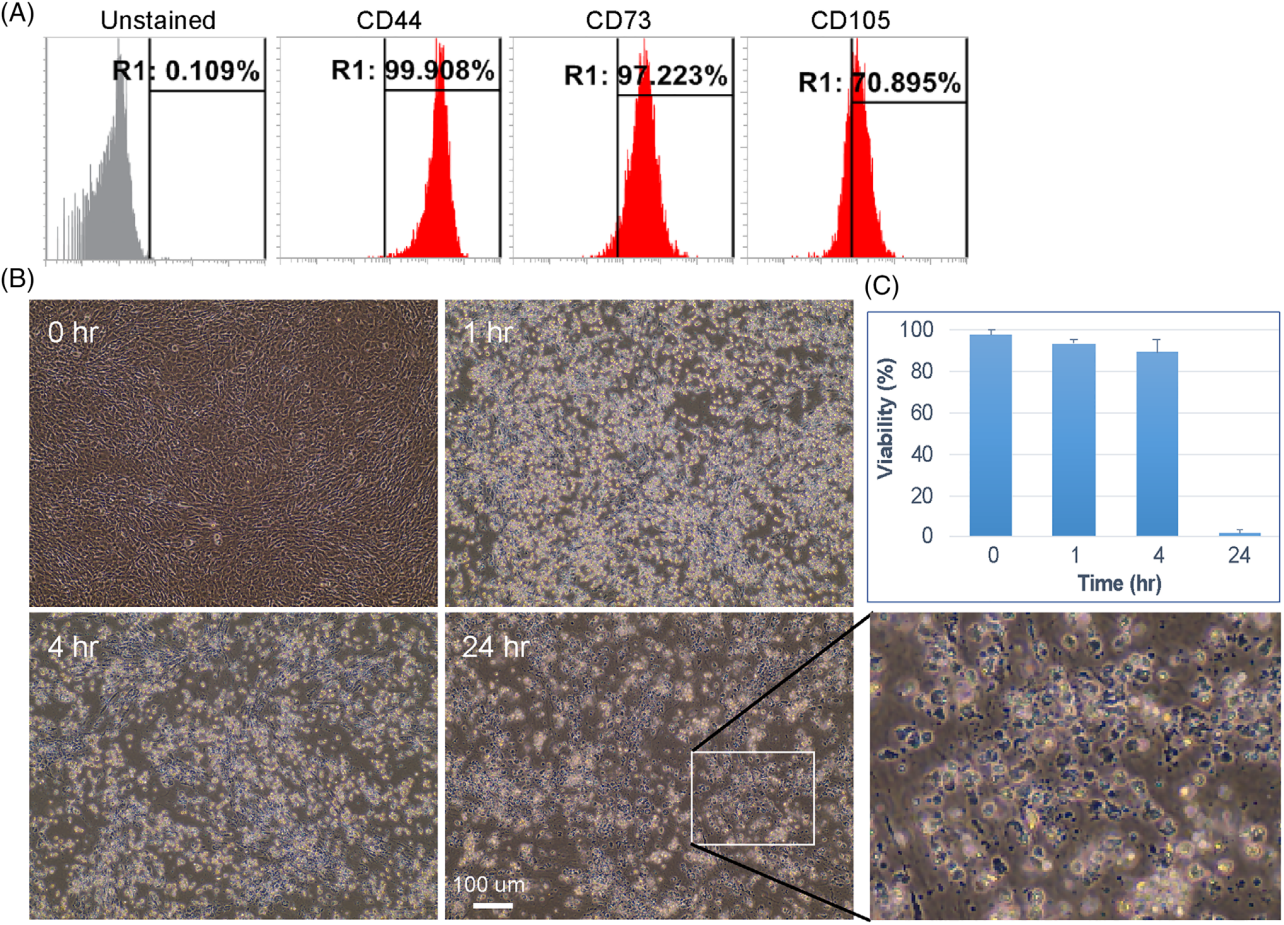
AP1903 effectively eliminates CAG‐iCASP9 induced pluripotent stem cells (iPSC)‐derived mesenchymal stem cells (MSCs). A, Flow cytometry for marker expression in CAG‐iCASP9 iPSC‐derived MSCs. B, Representative cell morphologies of CAG‐iCASP9 MSCs after treatment with AP1903. A blow‐up image showing no survival cells left after 24‐hours treatment. C, Quantifications of killing efficiency. At 24 hours, the viability is significantly different compared with other time points

To study whether suicide gene engineering affects iPSC differentiation potential, we differentiated CAG‐iCASP9 iPSCs toward MSCs. We found that engineered iPSCs could still be differentiated into MSCs efficiently, and confirmed this using flow cytometry assay of the expression of surface markers CD44, CD73, and CD105 (Figure 4A). Upon treatment with AP1903, iCASP9 MSCs could be rapidly killed within 1 day (Figure 4B,C) at passage 7. These data suggest that installation of iCASP9 safety switch does not affect iPSC differentiation potential to MSCs and the safety switch is still working rapidly and effectively in the iPSC‐derived MSCs.

**FIGURE 5:**
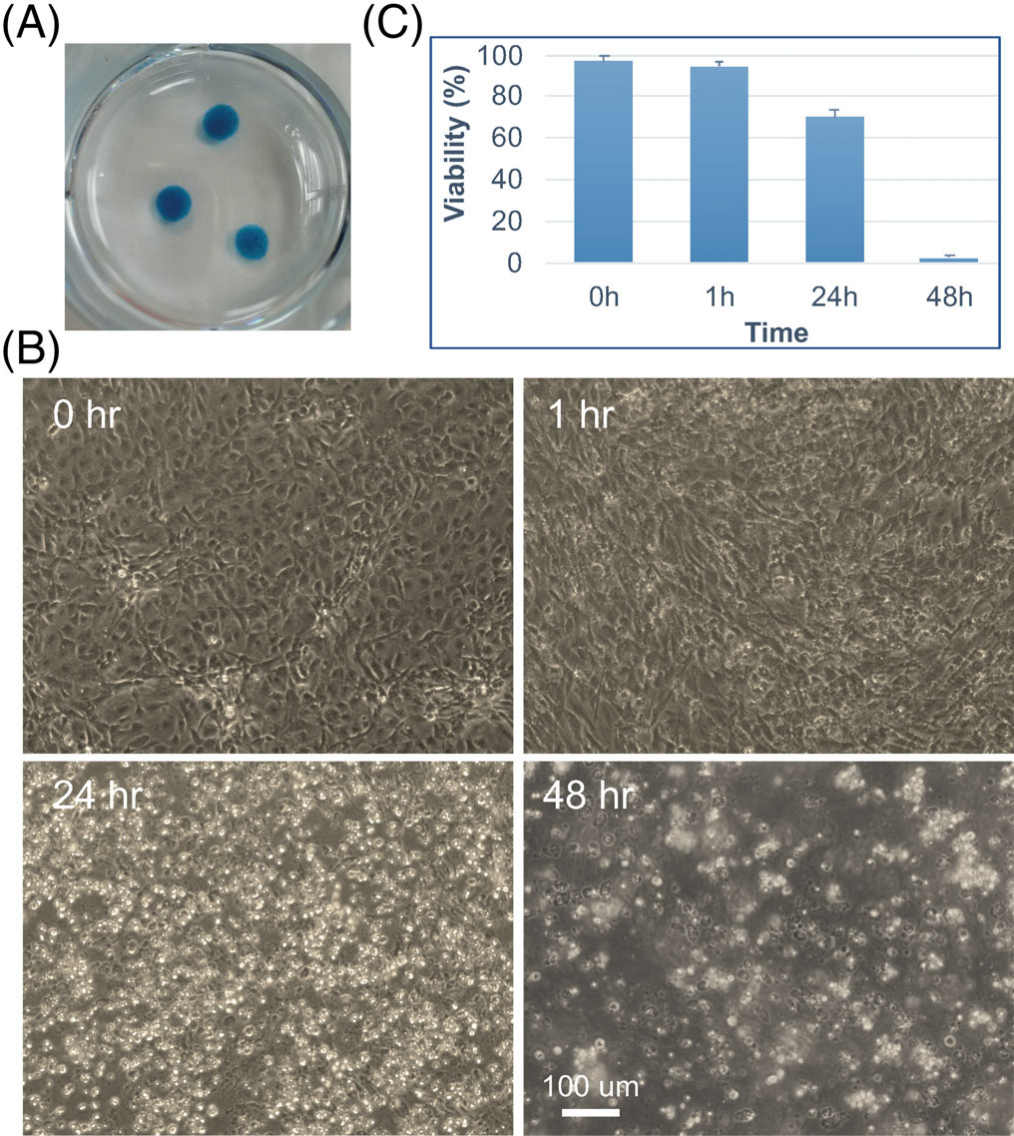
AP1903 effectively eliminates CAG‐iCASP9 induced pluripotent stem cells (iPSC)‐derived chondrocytes. A, Alcian blue staining on micromass chondrogenic differentiation from mesenchymal stem cells (MSCs). B, Representative cell morphologies of CAG‐iCASP9 chondrocytes after treatment with AP1903 at different time points. C, Quantifications of killing efficiency. At 24 and 48 hours, the viabilities are significantly different compared with other time points

We further differentiated these MSCs toward chondrocytes using a micromass differentiation protocol.^27^ After 1 week of differentiation in the chondrogenic medium, the micromasses were stained positive by Alcian blue (Figure 5A). This indicated that CAG‐iCASP9 iMSCs were able to differentiate and deposit glycosaminoglycans under proper chondrogenic differentiation conditions. Glycosaminoglycan deposition is one of the typical features of chondrocytes in micromass cultures.^30^ AP1903 treatment for 48 hours killed more than 97% of chondrocytes in micromasses (Figure 5B,C). These results suggest that the iCASP9 suicide gene system is still effective in iPSC‐derived chondrocytes.

### 3.4 AP1903 diminishes CAG‐iCASP9 iPSC‐derived teratomas in vivo

**FIGURE 6:**
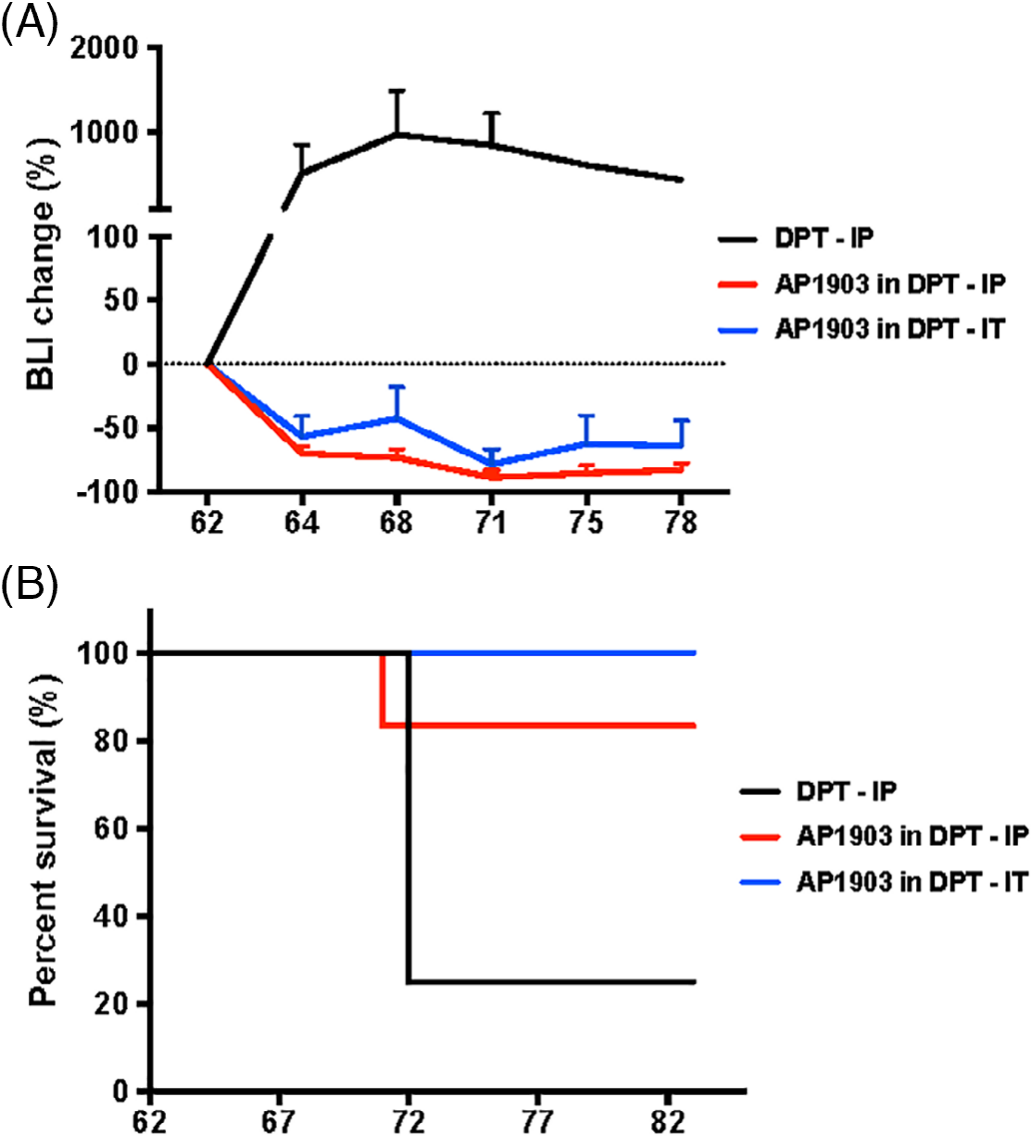
AP1903 rapidly clears CAG‐iCASP9 induced pluripotent stem cells (iPSCs)‐derived teratomas in vivo. A, Percentage of bioluminescence imaging intensity (BLI) change of tumors after AP1903 (in DPT) administration. Starting on day 62, the animals were injected with AP1903 in DPT. Both IP or IT AP1903 administration led to a significant shrinkage of teratomas as assessed by BLI change. By contrast, teratomas in animals dosed with vehicle only continued to increase in size. B, Survival curve. Mice were euthanized when tumor size reached 2000 mm^3^. One mouse was dead on day 9 in the AP1903 in DPT ‐ IP group, cause undermined. All other mice were euthanized at the termination of the experiment on day 83. DPT, 50% N,N‐dimethylacetamide/50% (90% PEG‐400/10% Tween 80); IP, intraperitoneal; IT, intratumoral

To further investigate whether CAG‐iCASP9 system works efficiently in iPSC‐derived teratomas, CAG‐iCASP9 iPSCs were injected into hind limb muscles in NSG mice and the resulting teratomas were characterized (Figure S4A). H&E staining revealed that all three germ layers were present in the teratomas (Figure S4B), suggesting installation of the suicide gene in the *AAVS1* locus does not affect the differentiation potential of human iPSCs. We then tested whether AP1903 could inhibit teratoma growth or eliminate them. AP1903 was administrated daily intraperitoneally (IP, n = 6) or intratumorally (IT, n = 6) from day 62 to day 67. Teratoma luminescence intensities in these two treatment groups decreased by more than 50% to 90%. In vehicle‐treated control mice (IP, n = 4), teratoma luminescence intensities increased by ∼1000% (Figure 6A). Eleven out of 12 mice in the AP1903‐treated groups survived at the end of experiment, whereas only one out of four mice survived in the vehicle control group, and others had been euthanized prior to the end of the study due to the large size of their tumors (Figure 6B). These data demonstrated that administration of AP1903 could shrink or even completely eliminate teratomas derived from CAG‐iCASP9 iPSCs in vivo (Figure S4C).

## 4 DISCUSSION

We successfully installed the iCASP9 suicide gene system into the genome safe harbor locus *AAVS1* in human iPSCs. The CAG promoter could more efficiently drive iCASP9 expression and kill iPSCs after AP1903 administration than could other promoters tested. iPSCs with CAG‐iCASP9 installed in both allelic loci of *AAVS1* were eliminated within 24 hours upon treatment of AP1903 administration, and no additional cell growth was observed after 10 days. CAG‐iCASP9 iPSCs retained the ability to differentiate into MSCs and chondrocytes in vitro and these derivatives could be efficiently killed when treated with AP1903. CAG‐iCASP9 iPSCs injected into NSG mice formed teratomas normally and the teratomas were significantly shrunk or eliminated after AP1903 treatment. Development of iPSC clones and subsequently derived cell types for clinical applications would involve removing antibiotic selection markers by Cre recombinase and further characterization of the cell lines by karyotyping and whole genome sequencing. The precise genome integration and improved cell elimination efficiency of the CAG‐iCASP9 suicide gene system described here may reduce safety concerns of human iPSC‐based therapies.

There are several known genome safe harbor sites including the *AAVS1*, *CCR5*, and *hROSA26*, but none of them have been tested clinically.^23^ The long‐term safety of disrupting these loci in human cells transplanted in humans remains to be determined. In this study, we precisely inserted iCASP9 into the *AAVS1* locus, with the endogenous *PPP1R12C* promoter, the EF1α promoter, a modified EF1α promoter, or the CAG promoter driving iCASP9 expression. The CAG promoter is a well‐known synthetic promoter that provides efficient and stable gene expression in a variety of cell types.^31^, ^32^ The CAG promoter can drive strong gene expression in human and mouse ESCs and their derivatives,^33^ whereas the activities of other constitutive promoters including CMV, UbC, EF1α and PGK gradually decreased during culture of human ESCs.^34^ Consistent with these previous studies, we found that among all of these tested conditions, only two copies of CAG‐iCASP9 were able to give high and stable transgene expression and efficient killing ability in iPSCs and their derivatives including MSCs and chondrocytes in vitro and teratomas in vivo. Previous studies also found that the EF1α promoter silencing was the main reason for incomplete killing ability of the iCASP9 system in iPSCs, although the iCASP9 iPSCs were not clonally derived.^19^, ^20^, ^22^ Since transgenes might be silenced and attenuated when human iPSCs differentiate into certain cell types, additional strategies to further enhance the safety warrant explanation. Examples include using two or more different promoters to drive suicide gene expression and/or installing suicide gene systems into two or more genomic safe sites.

The availability of alternate small‐molecule (eg, CID)‐based suicide safety systems for clinical cell‐based therapies is limited. The authors of this article emphasize the need for design and development of new systems of “keys” and “locks.” Rational in silico design of new CIDs (ie, the “keys”) and variations of the iCASP9‐fusion protein “locks” should yield new combinations that can be tested for safety and efficacy for use in future iPSC‐derived cell products. When considering iPSC‐based tissue implants or iPSC‐derived cell therapies designed to engraft in the human body and persist for years, aspects of long‐term safety must be considered. Importantly, the following points should be addressed:

- A given patient cannot receive different iPSC‐derived cell therapeutics intended for long‐term residency in the body that contain the same “key/lock” suicide system. The current lack of alternatives to AP1903 and the iCASP9‐FKB506 fusion system hinder the use of more than one cell type at a time in a given patient.
- Although iPSC‐derived cell products for clinical use are screened for oncogenic events that could potentially drive later tumor formation, it must be recognized that no screening system or combination of screening approaches can detect every single potential oncogenic driver in the cell products. Additionally, clinical cell products intended for long‐term engraftment and replication in humans are subject to the same age‐related damage as original, somatic cells and are not immune to future oncogenic transformation.
- The development of new CIDs or “keys” with different mechanisms of action might be accelerated by focusing on known biologically inert molecules and employing rational in silico drug design principles to custom design protein “locks” to fuse to the established iCASP9 suicide system.

As iPSC‐derived cell products enter clinical trials,^35^, ^36^, ^37^ the largest concern remains their potential oncogenicity. iPSCs are the ideal cell type in which to install safety mechanisms to eliminate cells that display undesirable effects after clinical administration. Because of the clonal nature of iPSCs, installing “suicide” systems (ie, inducible apoptosis), activated by otherwise biologically inert CIDs such as AP1903, represents a practical way to eliminate cells that later become troublesome. Our design and TALEN‐targeted precise installation of a CAG‐iCASP9 system into a genomic safe harbor locus in human iPSCs is a significant improvement in the efficiency and the safety on existing systems. Still, development of a diverse spectrum of precisely installed, high‐efficiency suicide safety systems activated by different small molecule “keys” is needed.

## 5 CONCLUSIONS

- The iCASP9 system was successfully installed the into the genomic safe harbor *AAVS1*.
- The CAG promoter induces strong and stable iCASP9 expression in iPSCs.
- Activation of this system with AP1903 leads to rapid killing and complete elimination of iPSCs and their derivatives including MSCs and chondrocytes in vitro, and iPSC‐derived teratomas shrank dramatically or even completely eliminated after administration of AP1903 in mice.
- Our data suggest significant improvements in safety and efficiency on existing iCASP9 suicide switch strategies.

## CONFLICT OF INTEREST

The authors declared no potential conflicts of interest.

## AUTHOR CONTRIBUTIONS

Z.‐D.S.: conception and design, collection/assembly of data, data analysis and interpretation, manuscript writing; J.T., L.W.: collection/assembly/interpretation of data; A.J.C.: conception and design, data interpretation, manuscript writing, final approval of manuscript.

## Supporting information


**Figure S1** EF1α core promoter and *PPP1R12C* endogenous promoter do not induce high GFP expression. (A) Representative images showing day 5 post‐nucleofection, puromycin‐resistant EF1α‐iCASP9‐2A‐GFP (Donor 1G) iPSC clones appeared but no GFP was detected. (B) 50 nM AP1903 treatment for 24 hours could not kill established clones from Donor 1G. (C) Representative images showing day 10 post‐nucleofection, puromycin‐resistant SA‐2A‐iCASP9‐2A‐GFP (Donor 2G) iPSC clones appeared but no strong GFP was detected. (D) 50 nM AP1903 treatment for 24 hours only killed some cells in the established clones from Donor 2G using the endogenous promoter.

**Figure S2** iCASP9 copy number in the established clones. Real time PCR was performed on genomic DNA to calculate iCASP9 copy numbers.

**Figure S3** Morphology of the cells after AP1903 treatment. Cells were smaller but more granulated by 24 hours than at 3 hours during AP1903 treatment.

**Figure S4** Teratoma assay. Representative image of a teratoma (A) and H&E staining showed three germ layers (mesoderm, endoderm and ectoderm) were formed in a teratoma (B). (C) Observations of teratomas in the mice. Definitions: Not identifiable: no teratoma could be identified within leg muscle, muscle appeared uniform throughout leg; Small: teratoma is identifiable from surrounding muscle, but is relatively small (no larger than a marker point); Medium: teratoma is identifiable, having taken over about half of the hamstring muscle (pea size or smaller); Large: teratoma and muscle can be distinguished, but teratoma has taken over majority of the hamstring muscle; Very large: Teratomas and leg muscle are indistinguishable, teratoma has completely taken over surrounding tissue in hamstring/quadriceps. IP ‐ intraperitoneal, IT ‐ intratumoral, ROA ‐ route of administration, DPT: 50% N,N‐dimethylacetamide/50% (90% PEG‐400/10% Tween 80).

## Data Availability

All data generated or analyzed during this study are included in this published article (and its supplementary information files).
