# Supplementary figures and images for: Precision installation of a highly efficient suicide gene safety switch in human induced pluripotent stem cells
Source: Stem Cells Transl Med. 2020 Jul 13;9(11):1378–88. doi: 10.1002/sctm.20-0007 (PMC7581441; doi:10.1002/sctm.20-0007)

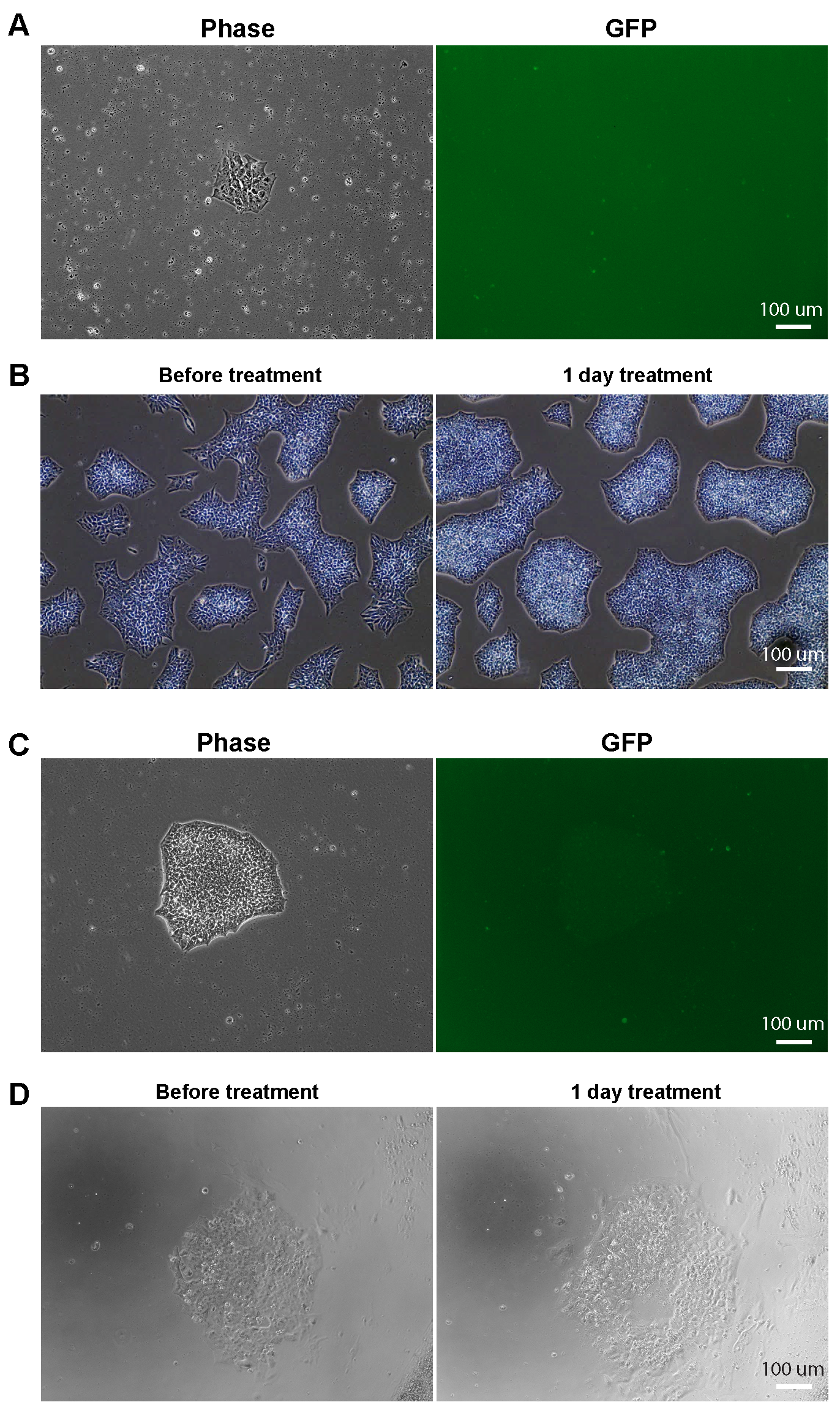

Supplement: Supplementary file 1 — Figure S1 EF1α core promoter and PPP1R12C endogenous promoter do not induce high GFP expression. (A) Representative images showing day 5 post‐nucleofection, puromycin‐resistant EF1α‐iCASP9‐2A‐GFP (Donor 1G) iPSC clones appeared but no GFP was detected. (B) 50 nM AP1903 treatment for 24 hours could not kill established clones from Donor 1G. (C) Representative images showing day 10 post‐nucleofection, puromycin‐resistant SA‐2A‐iCASP9‐2A‐GFP (Donor 2G) iPSC clones appeared but no strong GFP was detected. (D) 50 nM AP1903 treatment for 24 hours only killed some cells in the established clones from Donor 2G using the endogenous promoter. [file SCT3-9-1378-s001.tiff]

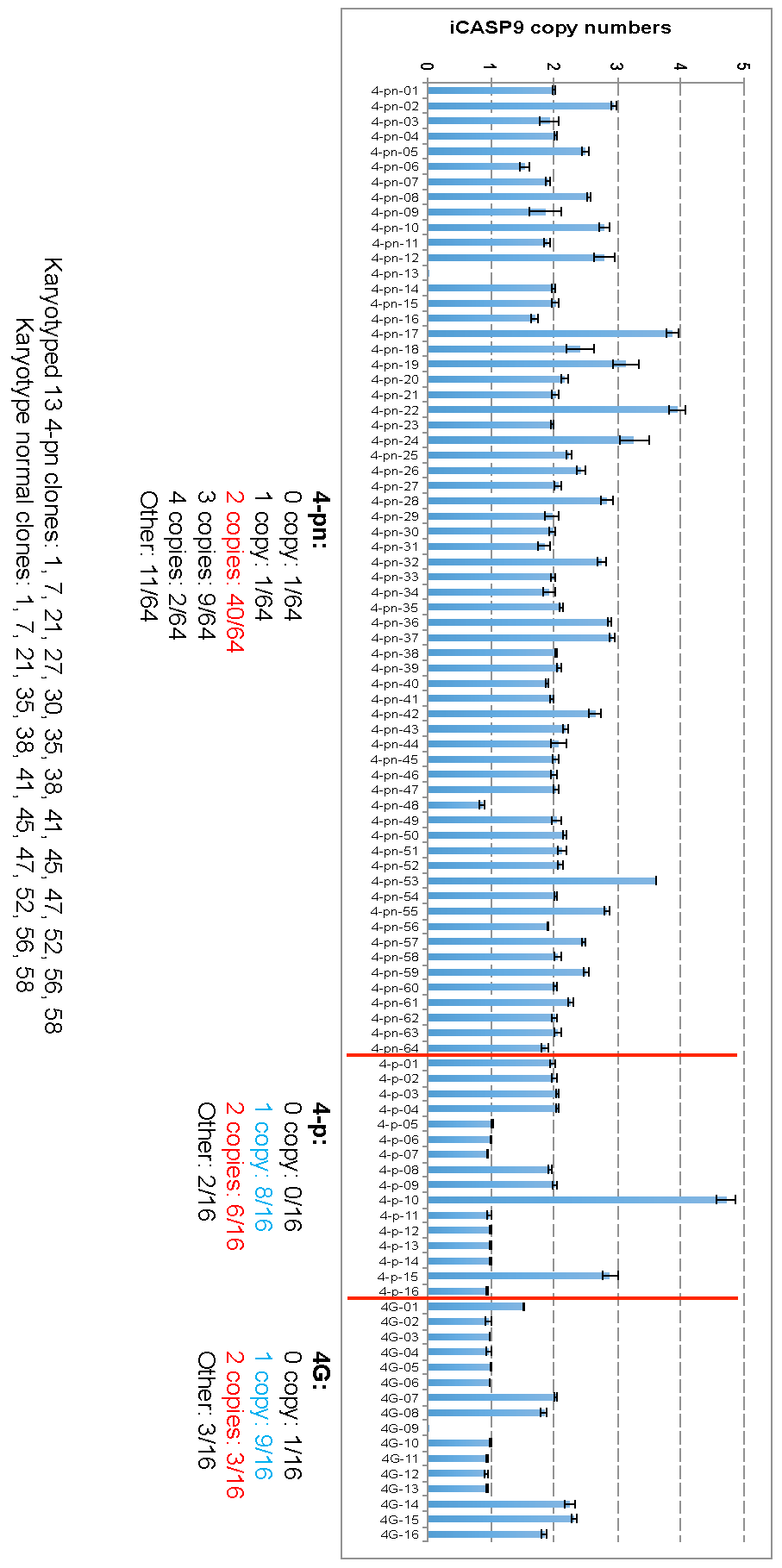

Supplement: Supplementary file 2 — Figure S2 iCASP9 copy number in the established clones. Real time PCR was performed on genomic DNA to calculate iCASP9 copy numbers. [file SCT3-9-1378-s002.tiff]

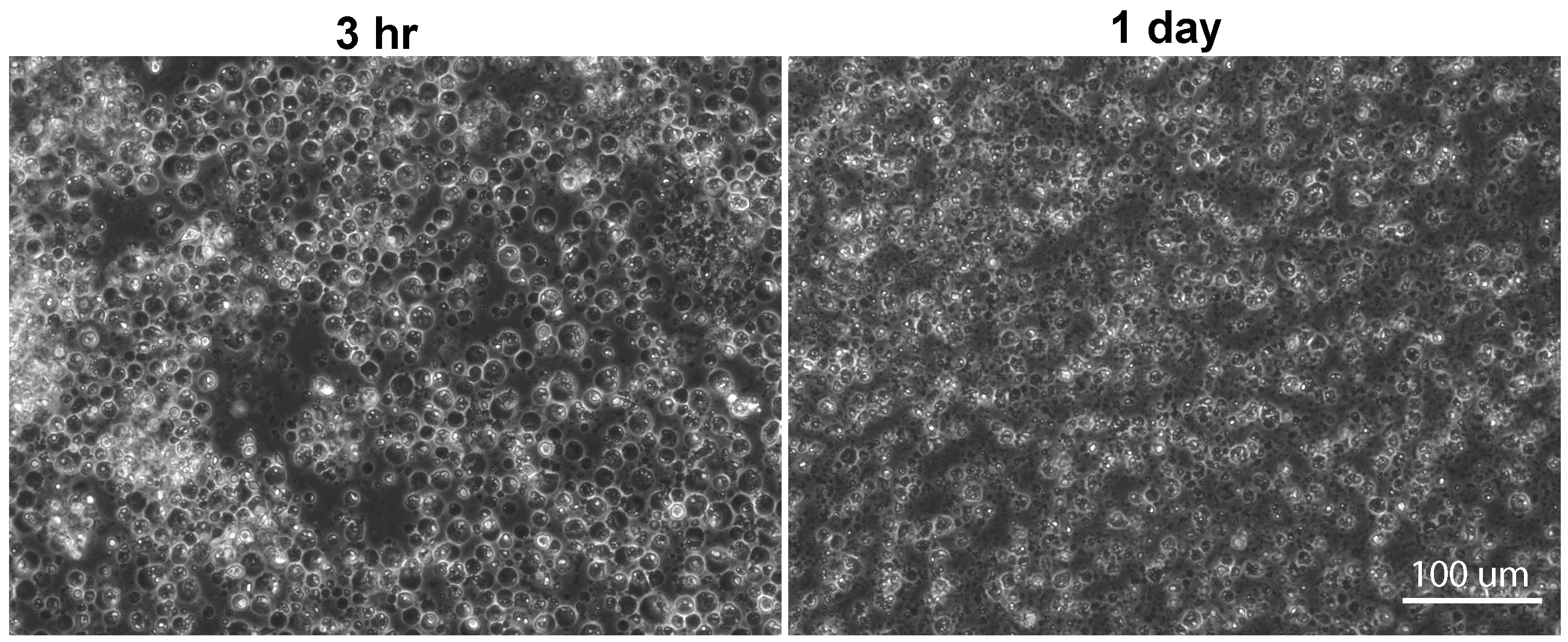

Supplement: Supplementary file 3 — Figure S3 Morphology of the cells after AP1903 treatment. Cells were smaller but more granulated by 24 hours than at 3 hours during AP1903 treatment. [file SCT3-9-1378-s003.tiff]

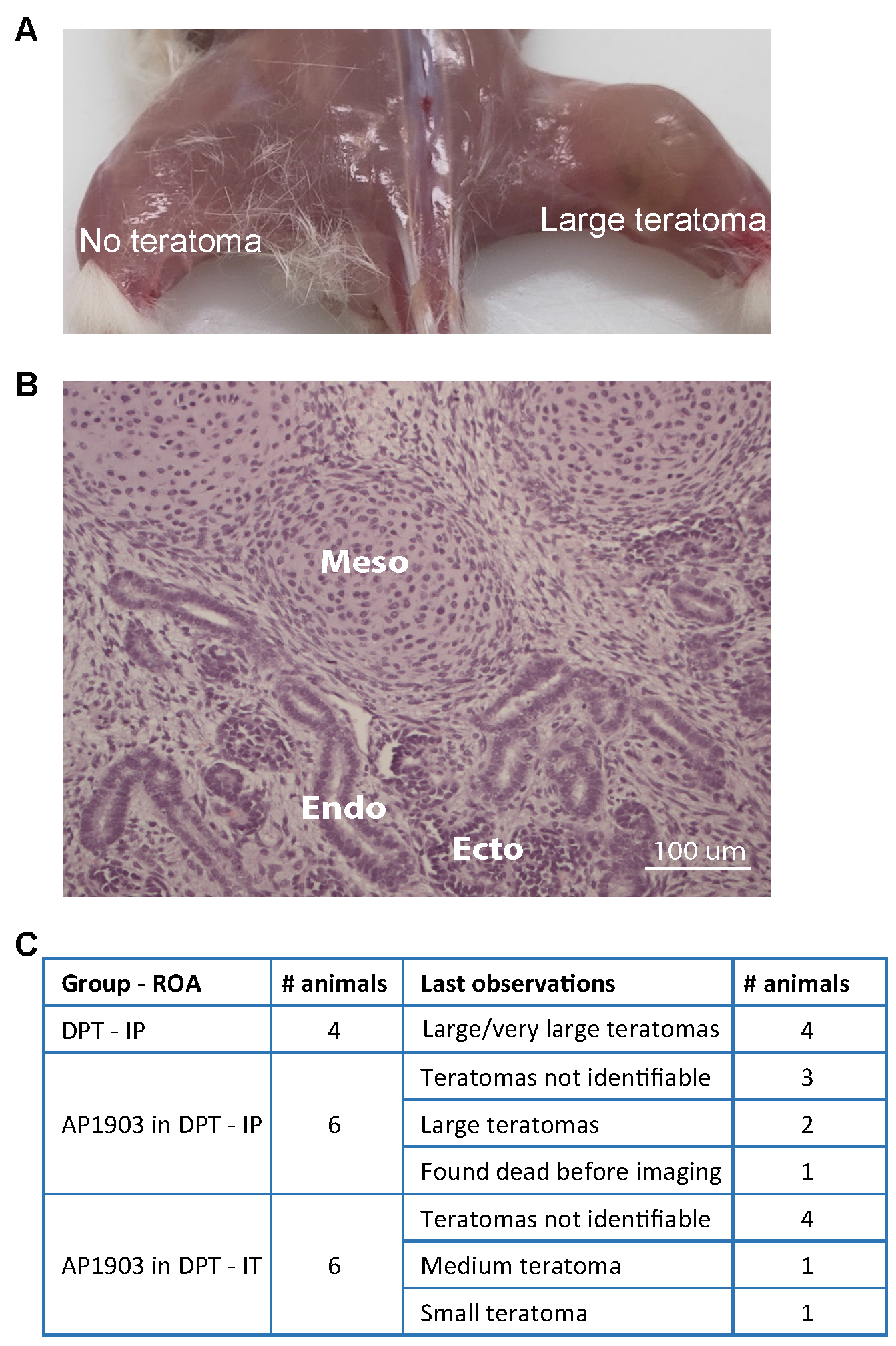

Supplement: Supplementary file 4 — Figure S4 Teratoma assay. Representative image of a teratoma (A) and H&E staining showed three germ layers (mesoderm, endoderm and ectoderm) were formed in a teratoma (B). (C) Observations of teratomas in the mice. Definitions: Not identifiable: no teratoma could be identified within leg muscle, muscle appeared uniform throughout leg; Small: teratoma is identifiable from surrounding muscle, but is relatively small (no larger than a marker point); Medium: teratoma is identifiable, having taken over about half of the hamstring muscle (pea size or smaller); Large: teratoma and muscle can be distinguished, but teratoma has taken over majority of the hamstring muscle; Very large: Teratomas and leg muscle are indistinguishable, teratoma has completely taken over surrounding tissue in hamstring/quadriceps. IP ‐ intraperitoneal, IT ‐ intratumoral, ROA ‐ route of administration, DPT: 50% N,N‐dimethylacetamide/50% (90% PEG‐400/10% Tween 80). [file SCT3-9-1378-s004.tiff]
